# Narratives for Positive Nature Futures in Europe

**DOI:** 10.1007/s00267-025-02123-3

**Published:** 2025-02-11

**Authors:** Alessandra D’Alessio, Claudia Fornarini, Nestor Fernandez, Anandi Sarita Namasivayam, Piero Visconti, Jeremy Dertien, Maria Hällfors, Martin Jung, Francisco Moreira, Louise O’Connor, Matea Osti, Laura C. Quintero-Uribe, Martina Marei Viti, Andrea Lauta, Henrique M. Pereira, Peter H. Verburg, Carlo Rondinini

**Affiliations:** 1 Global Mammal Assessment Program, Department of Biology and Biotechnologies, Sapienza University, Rome, Italy; 2 German Centre for Integrative Biodiversity Research (iDiv), Halle-Jena-Leipzig, Leipzig, Germany; 3 Institute of Biology, Martin Luther University Halle-Wittenberg, Halle-Wittenberg, Germany; 4 Institute for Environmental Studies, Vrije Universiteit Amsterdam, Amsterdam, the Netherlands; 5 Biodiversity and Natural Resources Program, International Institute for Systems Analysis, IIASA Laxenburg, Austria; 6 Nature Solutions Unit, Finnish Environment Institute, Helsinki, Finland; 7 CIBIO - InBIO Laboratório Associado, Universidade do Porto, Vairão, Portugal

**Keywords:** EU Biodiversity Strategy, IPBES Nature Futures Framework, conservation storylines, participatory scenarios, protected areas, nature restoration.

## Abstract

The Nature Futures Framework (NFF) is a novel approach for the development of positive scenarios centered on the relationship of nature and people, emphasizing biodiversity as part of the solution to environmental challenges across various spatial and temporal scales, explicitly addressing a plurality of values for nature. In this work, we describe the process that has led to the formulation of continental-scale positive narratives for conservation in Europe based on the NFF and its value perspectives (Nature for Nature; Nature for Society; Nature as Culture), through an expert group elicitation. We focused on 6 topics in the narratives: Nature Protection and Restoration; Forest Ecosystems; Freshwater Ecosystems; Urban Systems; Agriculture, and Energy. We analyze differences and similarities among the narratives across these topics. We develop three novel Nature Futures narratives for Europe with contrasting perspectives and priorities for the six topics. Within the EU socioeconomic trends and policy framework, common solutions that simultaneously tackle biodiversity conservation and instrumental and cultural Nature’s Contributions to People (NCP) provision emerged. This set of narratives may integrate preferences concerning EU-level conservation targets and plausible socio-ecological development pathways, supporting the modeling of positive scenarios for nature that can be crucial in guiding policy decisions towards recovery of nature.

## Introduction

The global biodiversity crisis has received increasing attention globally, but the actions have so far been insufficient to reverse the trend of declining biodiversity (CBD Secretariat 2020; IPBES 2019). In Europe, the EU Biodiversity Strategy for 2030 provides a framework for current and future conservation endeavors by setting clear targets and objectives that largely align with the Kunming-Montreal Global Biodiversity Framework (EC 2020a; KM GBF 2022). The strategy sets ambitious goals, including the expansion of protected areas (PAs) to reach a minimum of 30% spatial coverage for both land and sea. Importantly, at least one third of these areas should be managed under strict protection. In addition, the European Nature Restoration Law demands action to ecologically restore at least 20% of degraded land and sea areas within the EU, and support the recovery of ecosystems and species in synergy with area protection targets (EC 2022a). Yet, the long history of intensive exploitation of ecosystems in Europe and conflicts with other relevant socio-economic activities, such as agricultural, forestry, urbanization or energy production, makes the achievement of these policy targets challenging.

Achieving ambitious goals in the context of competing interests requires an integrated management approach that explores all relevant nature conservation values and options. Environmental change scenarios are valuable for nature conservation for investigating the potential impacts of different societal development pathways and policy choices on biodiversity and Nature’s Contributions to People (NCP), while also facilitating communication and involving multiple stakeholders in the process (Pereira et al. 2020). The widely used Shared Socio-Economic Pathways (SSPs) scenario framework integrates drivers such as demography, governance efficiency, inequality at both national and international levels, socio-economic advancements, institutional factors, technological advancements, and environmental conditions (van Vureen et al. 2014; O’Neill et al. 2014). However, scenarios based on SSPs typically do not take in consideration positive features specifically for nature and biodiversity, and are thus limited in their use for exploring different societal preferences concerning to the role of nature, as well as developing solutions, and related policies driving human socio-economic development (IPBES 2016; Saito et al. 2019; Pereira et al. 2020; Lundquist et al. 2021).

At the same time, it is increasingly clear that different preferences for nature exist, depending both on the relationship between people and nature, and nature management options (Dunn‐Capper et al. 2024; Carvalho Ribeiro et al. 2013; van der Wal et al. 2014). Recognizing the plurality of views of nature across people is important to democratize the management of landscapes, acknowledging tensions between stakeholders but also their perspectives on nature (Dotson and Pereira 2022). This richness of perspectives on nature is not currently represented in existing scenarios, with often only one “desirable” perspective for nature being considered in a given set of scenarios, which is often based on experts’ opinions rather than on a plurality of stakeholders’ perceptions (Rosa et al. 2017; Pereira et al. 2020).

To address the limitations within existing scenarios, the expert group on scenarios and models of the Intergovernmental Science-Policy Platform on Biodiversity and Ecosystem Services (IPBES) developed the Nature Futures scenario Framework (NFF) (IPBES 2023a). The NFF aims to support the development of positive scenarios centered on the relationship of people with nature across various spatial and temporal scales (IPBES 2023b; Kim et al. 2023). This framework incorporates different perspectives, all with nature at the center of the scenario design rather than just as an outcome, and allows the consideration of diverse value perspectives (Rosa et al. 2017; Pereira et al. 2020). NFF scenarios encompass three value perspectives that capture and cluster the many different preferences for nature across people (Mansur et al. 2022; Pascual et al. 2023), and can be represented as three corners of a triangle (Fig. S1). The Nature for Nature (NfN) perspective emphasizes the intrinsic value of nature, including preserving individual species and species diversity, habitats, ecosystems, natural processes, and the self-regulatory processes of nature. The Nature for Society (NfS) perspective focuses on the maximization of instrumental values, benefits, and services that biodiversity and ecosystems provide to people, including food provisioning, water purification, disease control. Finally, the Nature as Culture (NaC) perspective highlights the relational values between nature and people, where society, traditions, beliefs and emotions drive socio-ecological landscapes, such as silvo-pastoral landscapes (Bugalho et al. 2011; Zerbe 2022).

The NFF has been applied to assess preferences for nature in existing participatory scenarios (Quintero‐Uribe et al. 2022), to develop new scenarios, e.g., in a National Park in the Netherlands (Kuiper et al. 2022), in a rural landscape in northeastern Japan (Haga et al. 2023), and in urban management (Mansur et al. 2022). Recently, the framework has been adopted to explore how contrasting narratives would translate into land use scenarios for Europe by 2050 (Dou et al. 2023). However, the NFF has never been applied to formulate nature’s future narratives at a continental scale concerning the protection and restoration of Europe. These aim to integrate experts’ visions about EU conservation targets and plausible socio-ecological development pathways, thus supporting policy decisions towards recovery of nature.

Here we designed NFF narratives for Europe through a consultation with a group of experts from different sectors. While the group included a majority of nature conservation experts, experts from other sectors were included in the process, to develop narratives that cover the broader indirect effects of society and economy on nature. Experts were invited to join two participatory events, one in person and one online. The narratives reflect different perspectives that explore conservation and restoration priorities and policies. We aimed to answer the questions: what are possible contrasting positive futures for European landscapes? What are the common enabling conditions that need to be met for any of these positive futures to come to fruition? Through a participatory process, we gathered perspectives and priorities from the experts and formulated NFF narratives based on key topics: Nature Protection and Restoration, Forest Ecosystems, Freshwater Ecosystems, Urban Systems, Agriculture, and Energy. These topics emerged in the context of the current challenges for nature conservation to help envision a sustainable future for nature and society. The narratives can support integrated planning and land use modeling towards the achievement of EU policy targets, by supporting modelers in the field of conservation, and consequently assisting the EU Member States in developing an ecologically representative, resilient, and well-connected Trans-European Nature Network (TEN-N) (NaturaConnect 2024).

## Material and Methods

**Fig. 1:**
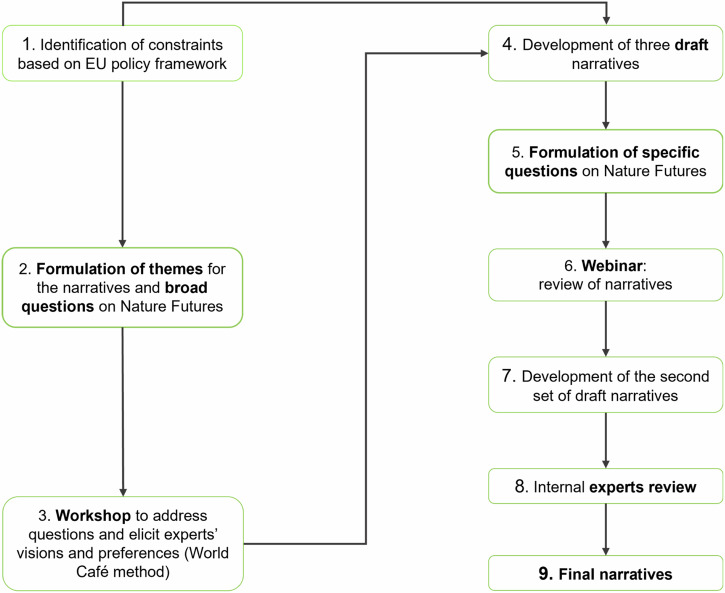
The process of development of the Nature Futures narratives for Europe

To develop the narratives aligned with the three NFF perspectives, representing the corners of the triangle (Fig. S1), we implemented the method from Pereira et al. (2020), into a sequence of nine steps (Fig. 1) (see Appendix 2 for further details).

**Fig. 2:**
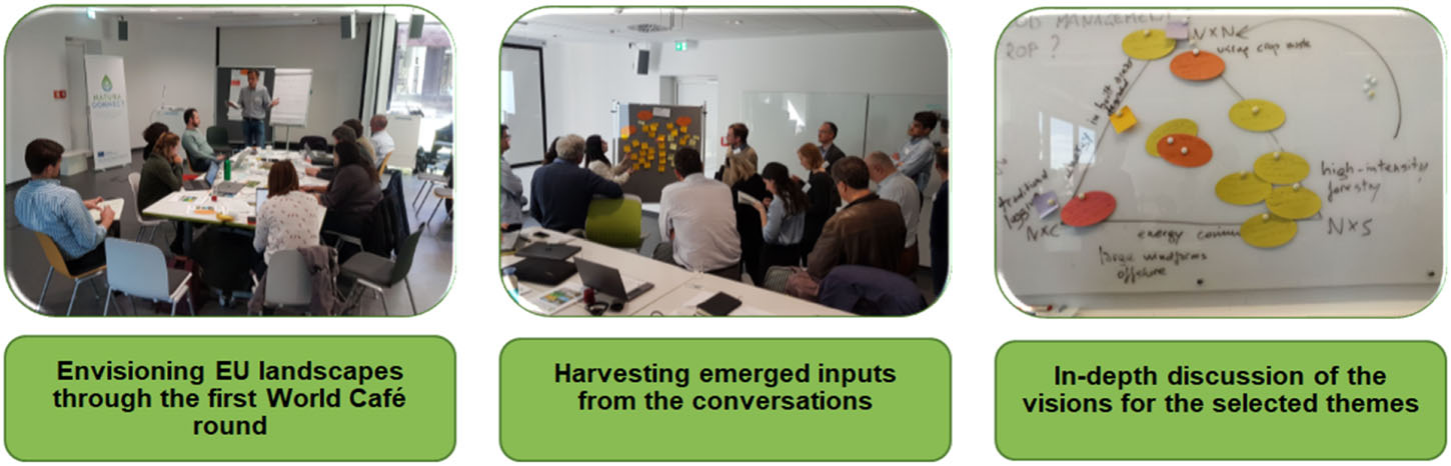
Session of Day 1 of the ‘Designing Nature Futures scenarios to support a Trans-European Nature Network’ workshop (NaturaConnect, CC BY 4.0). The World Café method has been used to facilitate discussion on several themes. Inputs emerged from the conversations have been collected as notes on post-it and later placed on each corner of the NFF triangle

**1)** We identified a set of EU assumptions, or ‘constraints’, that coerce the narratives about nature protection and restoration. We considered key EU legislation, regulations, objectives and strategic priorities as mandatory for all NFF narratives. These include the EU Biodiversity Strategy objectives for 2030, such as the expansion of PAs and strictly protecting one third of these areas; the implementation of multifunctional Green and Blue Infrastructure; and the Nature Restoration Law (EC 2022a). We also took into account the Common Agricultural Policy; the EU Farm to Fork Strategy (EC 2020b); the “No Net Land Take” by 2050 objective (EC 2016); and the European Climate Law (EC 2023b). **2)** According to the challenges and constraints facing Europe, we decided to address a preliminary set of themes and, based on them, we formulate a set of broad questions to be asked to people (Appendix 2.1). **3)** In a second phase, we identified key experts in the conservation field based on their influence in specific sectors of interest at the European level, and then we organized an in-person workshop with experts to elicit their perspectives on the future of nature protection. We held a three-day in-person workshop (Leipzig, Germany, 8-10 May 2023) with experts including several scientists of the NaturaConnect consortium with different expertise within the conservation sector (Tables S1 and S2). The workshop aimed to gather insights on the future of nature in Europe, using the World Café method for structured dialogues led by moderators (Brown 2010) (Fig. 2; Appendix 2.2).

The first World Café round, which focused on landscape changes, agriculture management, and conservation motivations, was facilitated by showing pictures of different European landscapes, selected according to the themes identified in the previous step. Participants moved between tables that represented the different corners of the NFF triangle to envision future European landscapes contrasting the three different NFF perspectives on nature. Subsequently, the discussion moved into the previously defined themes (Appendix 2.2). **4)** This visioning exercise was propaedeutic to develop the first draft of the narratives, by elaborating and revising the outcomes with moderators of each workshop’ session. After the workshop, indeed, we refined the three narratives “Nature for Nature”, “Nature for Society” and “Nature as Culture”, focusing them on six main recurring topics: Nature Protection and Restoration, Forest Ecosystems, Freshwater Ecosystems, Agriculture, Urban Systems, and Energy (Appendix 2.3). **5)** Since gaps concerning preferences and different perspectives emerged, particularly on the Nature Protection and Restoration topic, we defined additional questions on nature futures to improve the narratives (Appendix 2.3). **6)** A draft version of the narratives was presented during a 2 h public webinar (4 July 2023). It served to harvest additional feedback and insights, through 15 interactive questions via Mentimeter (www.mentimeter.com), following each narrative presentation (Appendix 2.4). **7)** After the webinar, the most frequent remarks and new information were collected. Thus, both event inputs were analyzed and integrated to create a coherent second set of draft narratives. **8)** Finally, following a further review by the experts group of the NaturaConnect project, **9)** we developed a final set of narratives (Appendix 2.4).

We analyzed the main differences and commonalities across the narratives and we highlighted contrasts across the narratives concerning the six topics. Specifically, we analyzed some specific aspects involving the six topics that were key in distinguishing the NFF narratives: the dichotomy between land-sharing and land-sparing, the restoration approach, the importance of maintaining the integrity of freshwater resources, the human presence in protected areas, the level of forest management and gray infrastructures configuration. Reflecting the importance of these aspects in each narrative, we attributed each a gradient of preference from Minimum to Medium to Maximum.

## Results

The in-person expert workshop was joined by 41 participants from 13 European countries, including 13 external experts and 28 conservation scientists and practitioners from the NaturaConnect project (Tables S1 and S2). All participants represented institutions of the European environment conservation (95,4%), wildlife management (2,3%) and land use planning (2,3%) sectors.

The webinar brought together a group of 115 participants from 18 countries, all European except one. The sectors the experts belong to are distributed as follows: nature conservation (54%), land use planning, (13%), forestry (9%), social science (8%), policy and law (5%), urban (3%), marine (2%), agriculture (1%), tourism (1%) and other sectors (4%). Based on the webinar participants’ responses (60%), 80% belonged to nature conservation governmental or non-governmental organizations. However, it should be noted that 35% of participants who participated in the webinar their affiliated entity and sector remained unknown.

**Fig. 3:**
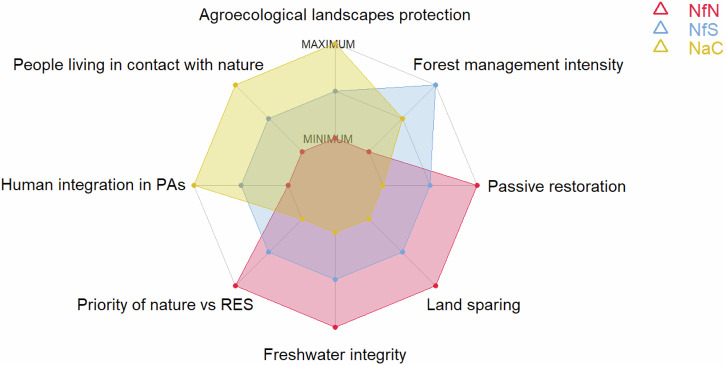
Spider diagram showing the main differences among the Nature Futures for Europe. The red, blue, and yellow polygons represent NfN, NfS and NaC, respectively. Axes represent a gradient measured on an ordinal scale from Minimum to Medium to Maximum. This gradient reflects experts’ visions for all NFF corners, on topics selected for drafting the narratives (Nature Protection and Restoration, Freshwater Ecosystems, Forest Ecosystems, Agriculture, Urban Systems, and Energy)

Through the experts’ consultation, we designed three narratives that describe different nature futures in Europe, one per each corner of the NFF triangle: Nature for Nature (Box 1), Nature for Society (Box 2) and Nature as Culture (Box 3). Below we highlighted the differences among the narratives, stressing the main aspects differentiating the Nature Protection and Restoration topic across them (Table 1), and the commonalities (Figs. 3 and4).

**Table 1:** Summary of the Nature Protection and Restoration topic

Subtopic	Nature for Nature	Nature for Society	Nature as Culture
Priority objectives for restoration and PA expansion and management	Emphasis is on ecological integrity and resilience. Irreplaceable and particularly vulnerable species and ecosystems receive high priority.	Emphasis on Nature’s Contributions to People (NCP) provisioning and associated species and ecosystems.	Emphasis on cultural landscapes, including high nature value farmland and associated species.
Priority for strict protection	Preserving sites with high ecological integrity (at least 10%), where no management and no intervention is carried out.	Preserving ecosystems for which the processes and functions associated with NCP depend on minimal disturbance.	Preservising culturally relevant species and ecosystems which require minimum disturbance.
Spatial priority for restoration and PA expansion	In areas of conservation concern to avoid anthropic disturbance.	In areas where NCP demand and supply is high.	In areas accessible to people.
PA size	Both large PAs to sustain ecosystems, and smaller PAs as part of corridors and stepping stones between larger areas.	Both large PAs for NCP related to large ecosystems (e.g. flood regulation), and small PAs for pollination around crops, air quality regulation and pest control.	Both large PAs to protect large traditional landscapes, small PAs to protect pocket parks inside cities.
Green and Blue Infrastructures role	To improve the structural and functional connectivity for all species.	To improve connectivity that supports NCP in peri-urban landscapes and across cultivated land.	To improve connectivity for symbolic species and cultural landscapes, in agro-ecological areas with hedgerows and natural patches, and cities.
Human Activities inside PAs	Only human activities in line with biodiversity conservation objectives are allowed.	Human activities/ intervention related to Nature’s Contributions to People are allowed.	Cultural human activities are allowed.

The topic is focused on different subtopics for each narrative (in column): conservation priority, details on the Protected Areas (PAs) aim and use (e.g., human activities, strict protection, location, size), Green and Blue Infrastructures role, and the restoration strategy

**Fig. 4:**
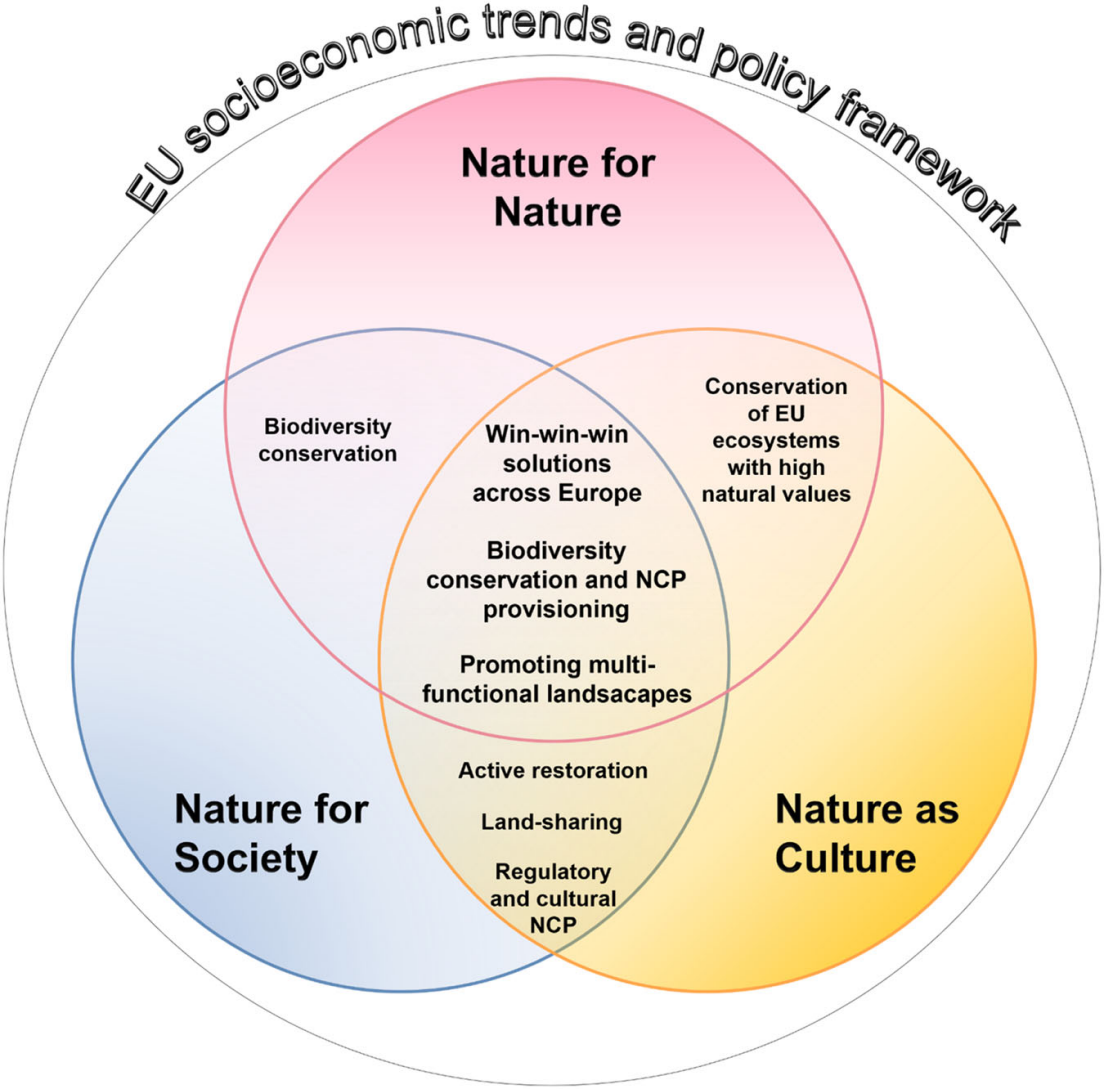
Venn diagram showing the commonalities among the Nature Futures for Europe. Overall, win-win-win solutions, biodiversity conservation and Nature’s Contributions to People (NCP) provisioning, and the promotion of multi-functional landscapes are envisioned for all NFF corners

Box 1 Nature for Nature (NfN) In the NfN narrative, the value of nature is intrinsic and independent from any direct benefits that people may gain from nature. The protection and restoration of the ecological integrity of ecosystems are therefore key priorities in this narrative and thereby land sparing approaches are pursued. Strict protection is envisioned for natural areas to preserve the integrity and resilience of nature within the European protected area network. Conservation focuses on sensitive and irreplaceable species and habitats. PAs are located in areas far from people, and human activities are also minimized in PAs as access to these areas is restricted.

Primarily, large protected areas that can sustain self-regulated ecosystems are established, but smaller protected areas also can play a complementary role as part of corridors and stepping stones between larger areas, especially in highly fragmented landscapes. Both structural and functional connectivity is improved for all species through Green and Blue Infrastructures. Restoring and ensuring the connectivity of PAs is a priority pursued to help recover the characteristic ecological flows of undisturbed ecosystems. Restoration of connectivity in freshwater ecosystems is essential in this narrative and obsolete dams are removed for this purpose. Natural forest dynamics is promoted, thus enhancing both structural and functional complexity and natural regeneration and turnover. Forest harvesting is reduced to a minimum, especially in old-growth forests and in strictly protected areas. To leave space for nature conservation, high-intensity agriculture is maintained and integrated with NBS to some extent, to maximize production without expanding agricultural land, and high-rise compact cities development are deemed desirable. To avoid wildlife mortality and disturbances, the impacts of renewable energy production are minimized by placing the energy plants in already degraded areas and high-intensity agricultural landscapes with low biodiversity values, also excluding buffer zones around PAs and other sensitive conservation areas.
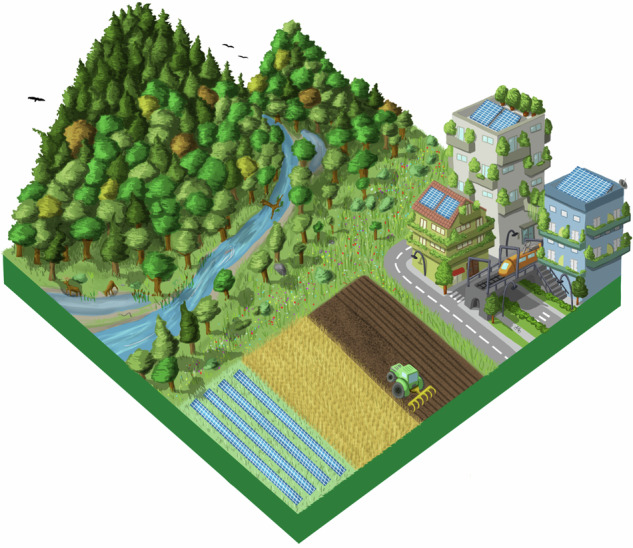

Box 2 Nature for Society (NfS) In the NfS perspective, emphasis is placed on the instrumental value provided to people. As a result, ecosystems are protected and restored with the aim of boosting the provisioning of NCP. To allow this provisioning, PAs are located where both NCP supply and demand are high and human activities are moderate. The network encompasses both small and large PAs since size depends on the services they provide: large PAs are selected for water and flood regulation and carbon sequestration; smaller PAs are established in proximity to people and supply pollinator habitats around crops, air quality regulation and pest control. Species conservation is a priority mainly when it is associated with the supply of a specific NCP. Ecosystems for which the processes and functions associated with NCP depend on minimal disturbance are strictly protected. Ecological corridors are designed and restored taking into account their capacity to provide multiple benefits to people, especially in peri-urban landscapes and across cultivated land through Green and Blue Infrastructures (EC 2019). Overall, active management and restoration approaches are used to prevent natural hazards (such as fire and flood risk) or reverse their impacts, promote carbon sequestration and sustainable timber extraction in forests, guarantee good water quality and supply, and ensure wild fish supply in freshwater ecosystems, where dams are managed to have minimal impacts on biodiversity. Moderate land sharing is necessary for providing NCP in agriculture and urban areas. High intensity agriculture and farming are away from areas of conservation concern and integrated with NBS, to increase biodiversity that leads to a better provision of NCP. Agroecological landscapes are maintained for species (e.g. farmland birds) and habitats of high conservation interest, such as *Dehesa*, which is an extensive agrosilvopastoral system typical of the Iberian peninsula (Parra-López et al. 2023). Moderately compacted urban areas are planned to facilitate beneficial contact between society and natural features. Although the provision of renewable energy is given priority over nature, plants are placed within agricultural landscapes to reduce the overall impact on biodiversity and its associated NCP.
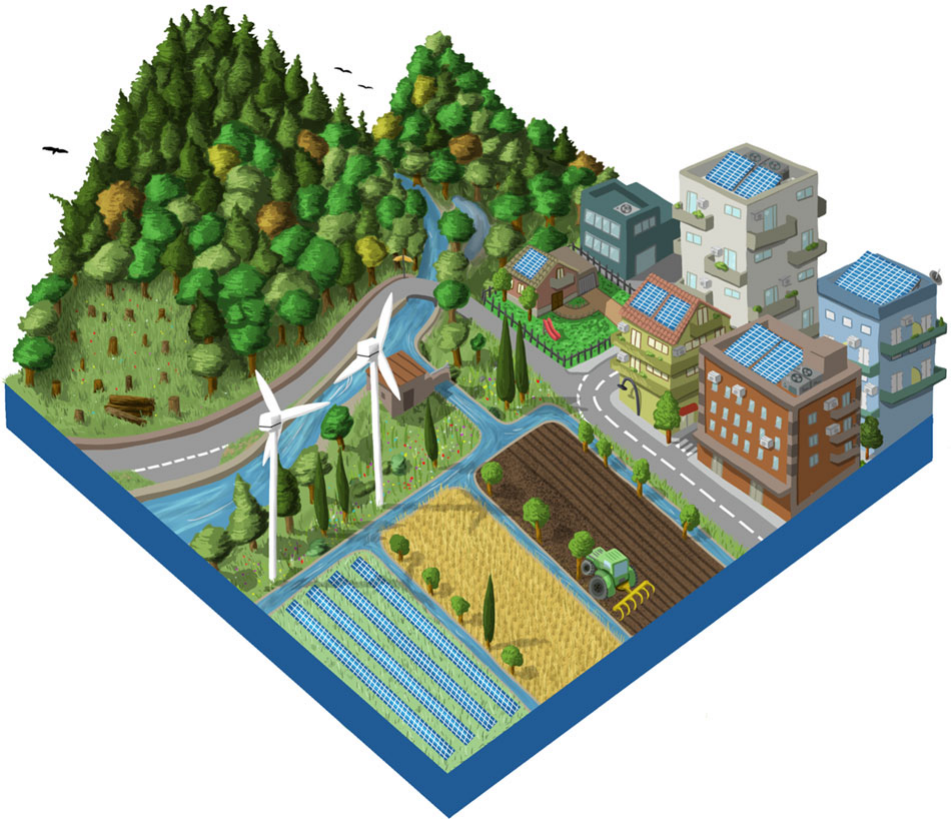

Box 3 Nature as Culture (NaC) The NaC narrative focuses on the relational values for nature, expressing personal and collective emotional connections that people have with nature. Therefore, human activities and presence within nature are tolerated more in this narrative than in the others, and PAs are preferentially located near the human population to enable people’s access to nature. Strict protection focuses on culturally relevant species and ecosystems which require minimum disturbance. Overall, conservation efforts address species and habitats associated with culturally important activities, such as fishing or hunting, and the expansion of PAs aims to meet conservation objectives that preserve culturally valued species (e.g., migratory birds and fish, charismatic species), habitats (e.g. agroforestry systems, hay meadows), ecosystem services and human-modified systems with high natural value (e.g. heritage landscapes, agroforestry systems, hay meadows) (Halada et al. 2011). These are done through initiatives such as UNESCO Man and Biosphere reserves (MAB) (Reed 2019). Thus, traditional land use practices and experiences that connect people to specific landscapes are prioritized in large PAs (e.g., Farm to Fork initiatives, wine routes, transhumance of livestock, high nature value farmland, biodiversity-friendly farming, pilgrimage routes, hiking and enjoyment of nature). Small PAs aim to protect pocket parks inside cities. The traditional cultural landscapes and habitats are restored, and their connectivity is improved, with an additional aim to bring nature back to highly degraded areas, cities and agroecological areas through Green and Blue Infrastructures. Forests are managed by prioritizing tree species with high cultural value. Ancient trees and other natural monuments are preserved. Freshwater ecosystems with a historical and cultural role, or those that are important for emblematic species, are also protected and restored, removing obsolete dams unless they have cultural importance. In rural areas with high conservation and cultural value, extensive and traditional agricultural practices, often integrated with NBS, are revitalized. These activities enhance the connection between nature and people that prefer living in rural areas. Less consideration is taken of the impacts of renewable energy infrastructure on nature, concealing them from humans in order to preserve the aesthetics of the landscape.
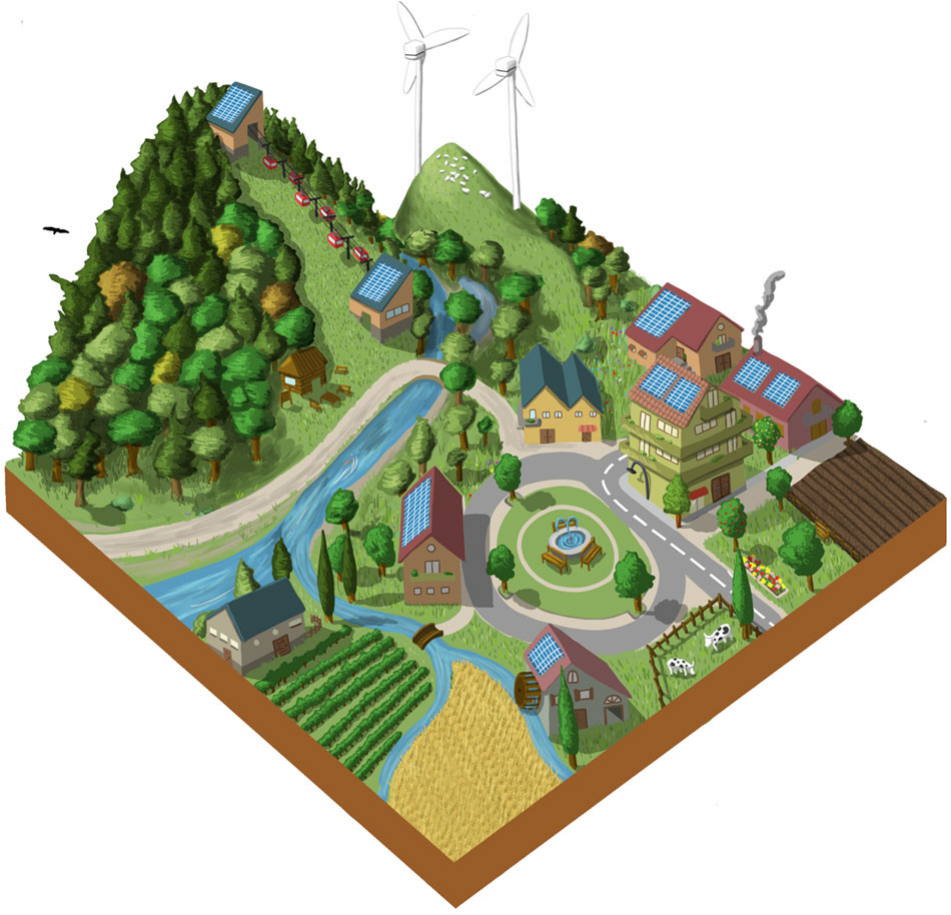

### Differences among the narratives

The main difference among the narratives are the preferences towards the land sparing or land sharing approach to protect and restore nature, which shape the associated outcomes of Agriculture, Urban System, Forest Ecosystems and Energy topics (Fig. 3).

In the NfN perspective, land sparing is preferred to save space for nature (Box 3), whereas, in NaC, land sharing is favored to allow the cultural use of the landscapes (Box 1). NfS requires a moderate gradient of land sharing to provide NCP (Box 2). Thus, human infrastructures and agricultural systems are compacted at their maximum in NfN and located away from areas of conservation concern, and they are moderately compacted in NfS, leading people to live far from nature. Conversely, in NaC, anthropogenic landscapes are integrated with nature, connecting people to nature more closely, and infrastructures are placed in isolated areas to avoid culturally important places and landscapes. The protection of agro-ecological landscapes, which lies on the enhancement of biodiversity and related ecosystem services to support traditional agricultural production, is maximized in NaC and moderate in NfS, while it has low importance in NfN.

In NfN, ecological integrity and connectivity have priority over renewable energy sources (RES, e.g. wind and solar farms). In contrast, nature has low priority over the impacts of RES plants in NaC, while being moderate in NfS (Fig. 3). Also, integrity of freshwater ecosystems is central to the NfN narrative for species connectivity and ecological flows, and less relevant in NfS and NaC, where flood regulation and recreational activities related to freshwater are given more importance.

In NfN, passive restoration was preferred over active restoration, with forests left to regenerate naturally, enhancing the complexity of forests. In NfS and NaC, an active restoration approach was preferred, with forests actively managed by local authorities to maximize NCP and biodiversity in NfS, or managed by local communities to enhance cultural activities in NaC. In the NfN perspective, minimal human activities in PAs were foreseen, because access to these areas should be limited. They were expected to be moderate in NfS and more intensive in NaC, where PAs should be located near human settlements to improve accessibility (Fig. 3). The implications of the three narratives for PA expansion and management, and for restoration are summarized in Table 1:

### Commonalities

Some common concepts emerged across the narratives, since they were all based on the 2030 EU Biodiversity goals, and included mutually beneficial solutions that address biodiversity conservation and NCP provisioning (Fig. 4).

Conserving biodiversity is recognised as a key achievement, also enhancing the related NCP provisioning. Conservation efforts can achieve multiple objectives for nature and people by enhancing ecosystem integrity and connectivity, and simultaneously ensuring the practical uses and cultural values of nature (Fig. 4). For example, restored natural areas along rivers may provide umbrella habitats and regulate flooding whilst also creating space for recreational activities.

The sustainable use of landscapes is a common solution; for instance sustainable forestry, is beneficial in terms of carbon sequestration, and availability of recreational areas, and it supports the maintenance of biodiversity, and its productivity, vitality, regenerative capacity, as well as the provisioning, over time, of material and regulatory NCP. Promoting sustainable multifunctional agricultural and forest landscapes is central especially in NfS and NaC, as it may support various functions concurrently. Additionally the sustainability of the landscapes is also ensured by implementing Nature-Based Solutions (NBS), which are cost-effective strategies, inspired and supported by nature, that enhance resilience while delivering environmental, social, and economic benefits (UNEP, 2022). Some examples of NBS are integrated pest management, regenerative farming and precision farming, woodland islets and hedgerows, green roof and walls, etc.

Infrastructure planning, including highways, railways, and renewable energy plants, are planned in a more sustainable way, minimizing impacts on species and ecosystems. Urban greening and gardening initiatives may reduce the human carbon-footprint and ensure environmental sustainability, NCP, biodiversity and connectivity.

## Discussion

Here, we formulated three NFF narratives in consultation with experts of the conservation sector. The involvement of scientists with expertise in different fields offered the advantage of addressing all the topics covered by the narratives and helped taking different perspectives into account.

Our narratives highlighted differences stemming from the three different sets of nature values that the NFF describes. The extent of land sparing or sharing emerged as the main aspect for teasing the three NFF perspectives apart (Kremen 2015). Land sparing is mainly useful to maintain the space allocated for spared reserves (Kremen 2015) as emerged in the Nature Protection and Restoration topic of the NfN narrative, focused on strict nature conservation (Box 1). The experts’ visions were less oriented toward this approach in NaC (Box 3), based on the general expectation that integrating people with nature and PAs can be beneficial in terms of recreation activities, carbon sequestration, pollination, livelihood, and biodiversity. Moreover land sparing has often been associated with higher species richness and it can be useful to achieve the conservation of the most sensitive species to human disturbance (Edwards et al. 2014; Cannon et al. 2019; Balmford 2021). The preferences toward land sparing are also reflected in other topics such as Agriculture, Urban System, Forest Ecosystems, and Energy. Land sparing in agriculture requires intensive cultivations, which are preferred also in NfN perspective, despite this approach could affect nature by reducing species’ richness at a local level (Balmford et al. 2005). Extensive agriculture systems, such as organic farming can increase biodiversity locally, because of the more heterogeneity of landscapes and can have beneficial impacts on the environment (Aldanondo-Ochoa and Almansa-Sáez, 2009; Gracia and De Magistris 2008), promoting pollination and biological pest control by avoiding mineral fertilisers and synthetic pesticides (Dimambro et al. 2018; IFOAM 2023; Senapathi et al. 2015; Tscharntke et al. 2021). However, the preference toward intensive agriculture even in NfN is based on the experts’ assumption that it has generally higher yield compared to extensive farming, leading to a reduction in land use with positive consequences on biodiversity at large scale. The protection of biodiversity overall is indeed a prerogative in modern agriculture, which is reflected at the European level in the EU Biodiversity Strategy (EC 2020a).

Concerning the Nature Protection and Restoration topic, the location and the aim of PAs are distinctive elements across the narratives. In the NfN perspective, PAs are located in areas where human presence and activities are minimized, to reduce the impacts on biodiversity. The experts’ consultation primarily led to the preference towards the establishment of large, protected areas that can sustain self-regulated ecosystems. Indeed, roadless and low-traffic areas are usually large, natural or semi-natural areas with no or few roads, and can substantially contribute to the preservation of biodiversity and NCP (Selva et al. 2015). However, in both NfN and NfS narratives, smaller PAs emerged to be a complementary solution, especially in highly fragmented landscapes. These small sized areas can be useful in targeting endemisms and species with narrow distribution ranges, and also to increase landscape connectivity and the provisioning of NCP (Volenec and Dobson 2020).

Despite the differences, some common concepts emerged across the narratives based on the 2030 EU Biodiversity goals and targets, including mutually beneficial solutions for biodiversity and NCP (IPBES 2016). Restoration efforts that enhance ecosystem integrity improve utilitarian functions such as water and air purification, pollination, climate change mitigation, and flood prevention, as well as the preservation of cultural values (Schindler et al. 2014; Zerbe 2022). We considered multifunctional landscapes crucial in the NfS and NaC narratives (Fig. 4). Their importance recur in different sectors, such as agriculture and forest ecosystems (Renting et al. 2009; Lindroth et al. 2012; Diez and García, 2012), as it has been pointed out across the NFF perspectives.

Efficient and carefully planned infrastructures, including renewable energy production and urban greening, are win-win-win solutions in all three positive nature futures (Fig. 4) to promote coexistence between humans and nature while minimizing negative impacts on species and ecosystems (Karteris et al. 2016). As envisioned in our NFF narratives, Europe is moving towards renewable energy sources (Bórawski et al. 2019), in order to adapt to the European Climate Law (EC 2023b). The expansion of renewable energy sources for Europe is essential to reduce net greenhouse gas emissions by at least 55% and reach carbon neutrality by 2050 (EC 2023a). Urban greening is fundamental for human mental and physical health (Lee and Maheswaran 2011) and for recreational and aesthetic appreciation (Veerkamp et al. 2021). Enhancing green areas is also relevant for cooling down cities, mitigating the effects of climate change, and reducing air pollution (Pauleit et al. 2020; Veerkamp et al. 2021). Community-based renewable energy and sustainable urban planning including zero-emission transportation, are examples of how to contribute to environmental sustainability, ecological connectivity, and improved human health simultaneously (Kammen and Sunter 2016).

Our NFF narratives are adapted to the European context, but consistent with the interpretation given to the same framework in other studies (Pearson 2016; O’Connor et al. 2021). However, compared with other narratives developed at global scale (Pereira et al. 2020), the priority for nature conservation in the NaC perspective did not just focus on the relational value assigned to certain areas —such as the UNESCO Man and Biosphere reserves (MAB) (Reed 2019)—, but also considered the historical value behind traditional practices associated with the European landscapes, such as vineyards or olive groves (UNESCO, SCBD 2014) and European Heritage sites (EC 2024). Narratives can be transformed into scenarios for environmental assessments, which are recognised as powerful tools for exploring how different pathways of societal development and policy choices could impact nature and the provision of NCP (Pereira et al. 2020). Some land-use and biodiversity models have been explored to determine whether it is possible to bend the biodiversity loss curve (Mace et al. 2018, Leclère et al. 2020). Although some scenarios demonstrated the feasibility of a positive outcome in this sense, there are still some limitations due to the challenges of further loss in several biodiversity-rich regions and threats, such as climate change, that have not been addressed (Pereira et al. in press). The NFF is a comprehensive approach that reflects diverse values and worldviews, and consequently helps identifying context-relevant interventions (Kim et al. 2024), by providing more flexibility in the development of scenarios. This has been done in Europe through scenario simulations which analyze synergies and trade-offs in land systems based on different value perspectives (Dou et al. 2023).

Our narratives can be interpreted and used as an additional layer that provides nuance and a representation of diversity in human-nature relational values to complement the macroeconomic assumptions of the SSPs/RCPs framework. At the same time, the development of the NFF scenarios is a step towards revising the commonly used set of SSPs dominantly based on assumptions related to climate change mitigation and adaptation efforts, with nature playing a central role alongside existing socioeconomic considerations (Rosa et al. 2017).

Narratives can serve as the foundation for exploring the integration of land use and nature conservation scenarios to achieve the global biodiversity strategy goals (Pereira et al. 2020; Kim et al. 2023), and in the perspective of policy design in Europe, to achieve EU conservation goals for 2030. Systematic conservation planning (SCP) has been used to identify areas of conservation and restoration priorities for people and nature at both global (Strassburg et al. 2020; Jung et al. 2021) and EU (O’Connor et al. 2021) levels. Our NFF narratives can therefore be translated in settings for land use modeling and SCP and used as inputs for identifying opportunities and constraints for conservation and restoration in Europe. It may inform ongoing and upcoming conservation planning research, such as the achievement of the TEN-N (EC 2020a), complementing the existing EU PA network in terms of species, habitats, and NCP, and to select suitable habitats within the future distributions of species and ecosystems in Europe.

## Supplementary information

Supplementary materials

## Data Availability

No datasets were generated or analysed during the current study.
