# Supplementary material for: Narratives for Positive Nature Futures in Europe
Source: Environ Manage. 2025 Feb 11;75(5):1071–83. doi: 10.1007/s00267-025-02123-3 (PMC12033173; doi:10.1007/s00267-025-02123-3)
Supplement: Supplementary file 1 — Supplementary materials [file 267_2025_2123_MOESM1_ESM.docx]

Supplementary materials

## Supplementary material 1: Nature Futures Framework (NFF) triangle


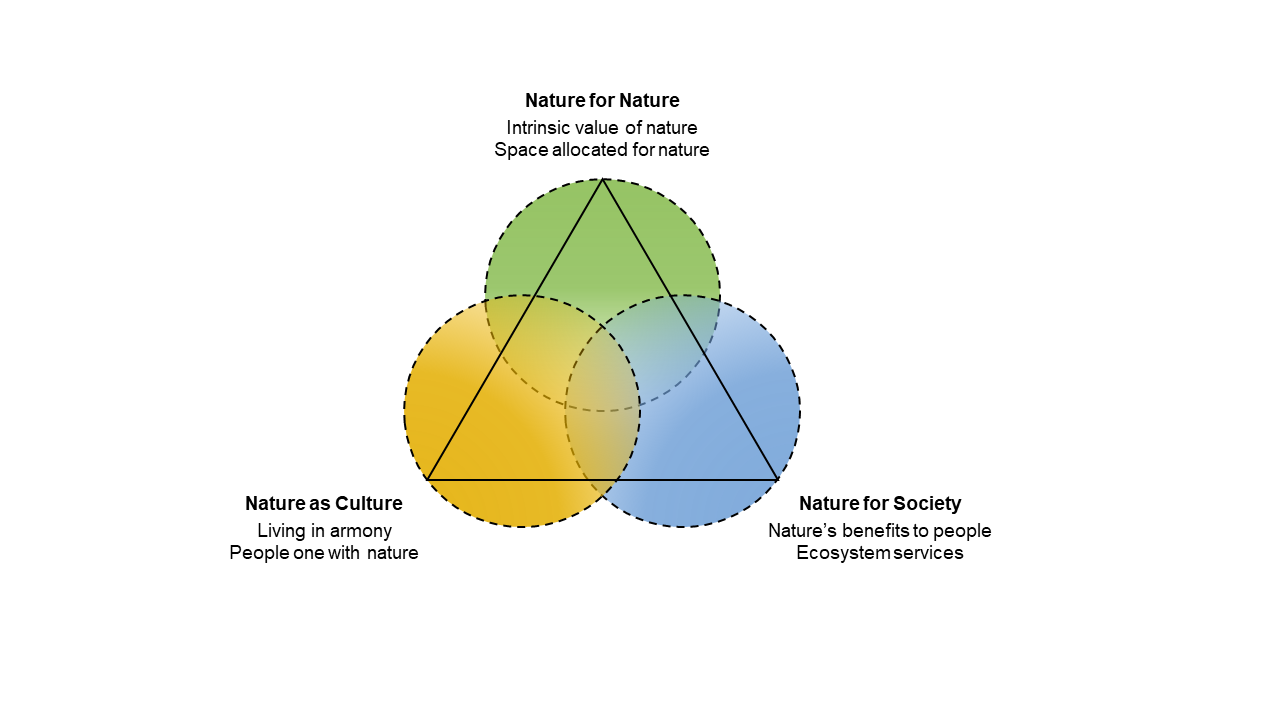


**Figure S1**: The Nature Futures Framework presents three value perspectives of nature in a triangle. Source: Adapted from Pereira et al., 2020.

## Supplementary material 2: Workshop and webinar

### Appendix 2. Supplementary materials and methods

The methodological approach consists of four main phases divided in nine steps that led to the elaboration of the draft narratives after the experts’ identification and the workshop. This was followed by the organisation of an online event to fill the gaps, the refinement of the narratives’ final version through a second elicitation stage, and the cross-narratives analysis.

The preparatory phase to the consultation consisted of  *1)* identifying a set of EU macroeconomic, social and legislative assumptions, or 'constraints', that coerce the NFF narratives; *2*) formulating questions on nature futures based on the constraints and identifying a set of priority themes to be discussed with the experts.

The second phase was about the selection of key experts within the conservation sector, based on their importance and influence across Europe, followed by the first consultation event i.e. *3)* an in-person workshop to elicit preferences around the themes and perspectives on the future of nature.

The third phase consisted of *4)* the elaboration of three draft narratives based on the outcomes of the workshop and *5)* the formulation of additional questions to be asked during a second event on nature futures.

During the fourth phase, *6)* the questions were presented during the second elicitation stage which was an online event, to obtain feedback on the draft narratives and fill potential gaps; *7)* Eventually, the narratives were then refined integrating the outcomes into a second set of draft narratives, and *8)* further reviewed by domain experts, which allowed us *9)* to develop the final set of narratives following the Nature Futures Framework.

#### Appendix 2.1 Preparatory phase to the experts’ elicitation: identification of constraints and preliminary themes for the narrative, and formulation of broad questions:

In order to pinpoint the constraints that coerce the narratives (**step 1**) we examined the EU legislation, regulations, goals, and strategic priorities that are essential for NFF narratives.We took into account the following key strategic goals:

- the increase in the coverage of protected areas (PAs) to a minimum of 30% for both land and sea, including 10% under strict protection. This expansion is intended to establish sufficiently large areas where vital natural processes can occur undisturbed (EC, 2022a). It also foresees the enhancement of the Natura 2000 sites and nationally PAs, improving the conservation status of species and habitats, and contributing to addressing future environmental changes (EEA, 2020);
- the European Nature Restoration Law (NRL), which targets the restoration of 20% of EU land. This initiative supports the recovery of ecosystems and species towards optimal ecological conditions (EC, 2022a). It also includes specific restoration actions for pollinators, river connectivity, forests, agriculture, urban areas, and marine ecosystems. Objectives encompass reversing the decline of pollinator populations, restoring rivers to a free-flowing state, achieving no net loss of green urban space by 2030, and increasing biodiversity in agricultural landscapes (EC, 2022a);
- the global goal of “No Net Land Take” by 2050, regarding the urban areas system. Assuming a linear progression in land take, achieving the EU's goal of reaching zero net land take by 2050 needs an annual reduction of 14 km2 from 2019 onward. Consequently, by 2030, the EU must decrease its yearly net land take to 282 km2; (EC, 2016)
- the EU Farm to Fork Strategy goals which were essential to set the constraints for the agriculture context. Such goals include transitioning 25% of agriculture to organic farming, reducing chemical pesticide use by 50%, and decreasing fertiliser use by 20% by 2030 (EC, 2020a);
- the European Climate Law (Regulation (EU) 2021/1119) that commits Member States to achieve the EU's climate goal of reducing emissions by at least 55% by 2030 compared to 1990. This involves measures such as increasing renewable energy sources to 32%, reducing fossil fuel biomass, planting three billion trees, and restoring carbon-rich ecosystems by 2030 (EC, 2023b).

Considering the main EU policy objectives, we examined a set of themes that are crucial for the future of nature and challenging for its conservation (**step 2**). After a brainstorming, which involved different domain experts, the following themes emerged: urban systems, forestry, freshwater ecosystems, green and blue infrastructure, habitats conservation and ecosystem restoration, agroecological policies, infrastructure development and renewable energies, and species conservation. We then formulated questions on the above mentioned themes, but also on specific priorities in PAs planning and connectivity design, to address experts’ visions of positive futures for people and nature in Europe, according to the different perspectives of the NFF. Some examples were: ‘What are the main changes happening in the landscapes in this Nature Futures (NF)?’, ‘What are the dominant changes in the management of agricultural areas?’, ‘Why do people conserve nature according to this NF vision?’, ‘What type of Nature-based Solutions do you expect according to this NF vision?’

#### Appendix 2.2 First elicitation stage: workshop to address questions and elicit experts’ visions

The broad questions were asked during the first experts’ consultation event, which was a three-day workshop, held in Leipzig (Germany) from 8 to 10 May 2023 (**step 3**). At this event, the scientists affiliated with the NaturaConnect project and with specific expertise were introduced as internal participants (Table S1).

**Table S1**: Internal participants of the Leipzig workshop “Designing Nature Futures scenarios to support a Trans-European Nature Network '' on 8-10th May 2023.

| **Stakeholders** | **Country** | **Organisation** | **Type of Institution** | **Expertise** |
| --- | --- | --- | --- | --- |
| Alessandra D’Alessio | Italy | Sapienza University | University | Green Infrastructure |
| Anandi Sarita Namasivayam | Netherlands | VU University | University | Land use change and ecosystem services |
| Barbara Herrero Cangas | Belgium | BirdLife Europe and Central Asia | NGO | European environmental policy |
| Carlo Rondinini | Italy | Sapienza University | University | Large-scale terrestrial mammal conservation, prioritisation and assessments |
| Clara Veerkamp | Netherlands | PBL | Research Institute | Nature-based solutions for urban sustainability challenges |
| Claudia Fornarini | Italy | Sapienza University | University | Status, trend, and conservation priorities of the world’s mammals |
| Daniele Baisero | UK | KBA Secretariat | NGO | Key biodiversity areas |
| Eloy Revilla | Spain | EBD - CSIC | Research Institute | Population and community ecology and conservation |
| Emmanuel Oceguera | Germany | iDiv | Research Institute | Geographic information systems |
| Filipe Dias | Portugal | CIBIO-BIOPOLIS | Research Institute | Conservation management of forests and riparian ecosystems under climate change |
| Francisco Moreira | Portugal | CIBIO-BIOPOLIS | Research Institute | Biodiversity in Agricultural and Forest Ecosystems and Impacts of energy infrastructures |
| Henrique Pereira | Germany | iDiv | Research Institute | Nature protection and restoration |
| Hildegard Meyer | Austria | WWF-CEE | NGO | Ecological connectivity & Green infrastructure |
| Jeremy Dertien | Germany | iDiv | Research Institute | Connectivity conservation |
| Jutta Beher | Austria | IIASA | Research Institute | Landscapes prioritization for protected area expansions and decision making for conservation |
| Laura Quintero Uribe | Germany | iDiv | Research Institute | Participatory biodiversity and ecosystem service scenarios in terrestrial systems |
| Louise O’Connor | Austria | IIASA | Research Institute | Climate and land use change scenarios for EU biodiversity and NCP conservation |
| Marek Giergiczny | Poland | University of Warsaw & iDiv | University and Research Institute | Environmental and behavioural economics |
| Maria Paniw | Spain | EBD - CSIC | Research Institute | Population Dynamics in Conservation Biology and Ecology |
| Maria Hällfors | Finland | Syke | Government administration or authority | ‪Climate change effects on biodiversity |
| Marit Schnepf | Germany | EUROPARC Federation | NGO | Integrated natural resource management and development in nature conservation |
| Martin Jung | Austria | IIASA | Research Institute | Ecological, socio-economic and environmental nexus |
| Martina Viti | Germany | iDiv | Research Institute | Conservation policy, biodiversity monitoring |
| Miguel Fernandez | Germany | iDiv | Research Institute | Environmental and sustainability sciences and climate change |
| Néstor Fernández | Germany | iDiv | Research Institute | Conservation policy, biodiversity monitoring and rewilding |
| Peter Verburg | Netherlands | VU University | University | Land change analysis, spatial analysis and modelling, human-environment interactions, human geography |
| Piero Visconti | Austria | IIASA | Research Institute | Conservation Planning for biodiversity |
| Sandeep Sharma | Germany | iDiv | Research Institute | Large-carnivore ecology and conservation biology |

Meanwhile, the other key experts involved in the workshop were considered external participants (Table S2).

**Table S2**: External participants who participated in the Leipzig workshop “Designing Nature Futures scenarios to support a Trans-European Nature Network '' on 8-10th May 2023.

| **Participant** | **Country** | **Organisation** | **Type of Institution** | **Expertise** |
| --- | --- | --- | --- | --- |
| Aija Kukkala | Finland | Syke | Government administration or authority | Coordination of national assessment for restoration act; spatial conservation planning |
| Bruna Campos | Belgium | EuroNatur | Charitable foundation | Forested and riverine areas; nature legislation and policy |
| Calin Ardelean | Romania | WWF | NGO | Wildlife monitoring; designing of ecological corridors; connectivity in the Carpathians |
| Cy Griffin | Belgium | FACE | International advocacy organisation | Interconnection between hunting, sustainable use and conservation |
| Francisco Guil | Spain | MITECO | Government administration or authority | Forest engineering, protected areas, rural development and rewilding |
| Frank Vassen | Belgium | European Commission | Government administration or authority | Environmental policy |
| Lea Suarez | France | OFB-MNHN-CNRS-IRD | Government administration or authority | Assessment of PA network in France |
| Lorenzo Ciccarese | Italy | ISPRA | Government administration or authority | Habitat and species conservation and sustainable management of agricultural and forested areas |
| Marita Böttcher | Germany | BfN | Government administration or authority | Connectivity, including EU-wide projects in relation to transport. |
| Markus Hedorfer | Italy | ECTP-CEU | No profit association | Urban Planning, Policy, Urban Design, Urban Regeneration, Sustainable Development |
| Michal Jecz | Poland | BULiGL | State enterprise | Forests and wetlands management |
| Sanja Fistric | Germany | Umweltschutz | Government administration or authority | Environmental protection |
| Víctor Cifuentes | Spain | C.H.Guadalquivir | Government administration or authority | Hydrological planning |

Considering the major importance of PAs planning and connectivity, we decided to dedicate Day 1 to the elicitation of diverse visions of NF for Europe, Day 2 to connectivity design, and Day 3 to planning PAs in the context of different NF.

During the morning session of Day 1, the NFF was presented to the participants, placing specific emphasis on highlighting the distinctions among the three nature futures perspectives. Following this, participants were asked to envision the future of European landscapes from the three perspectives. We asked participants to borrow some pictures of European landscapes which they were attached to and towards which they felt the need to reflect on. In addition, we showed different pictures of European landscapes related to the themes previously identified, to facilitate the discussions and elicit the experts’ visions about them, such as the configuration of urban systems or the renewable energy expansion (Fig. S2).
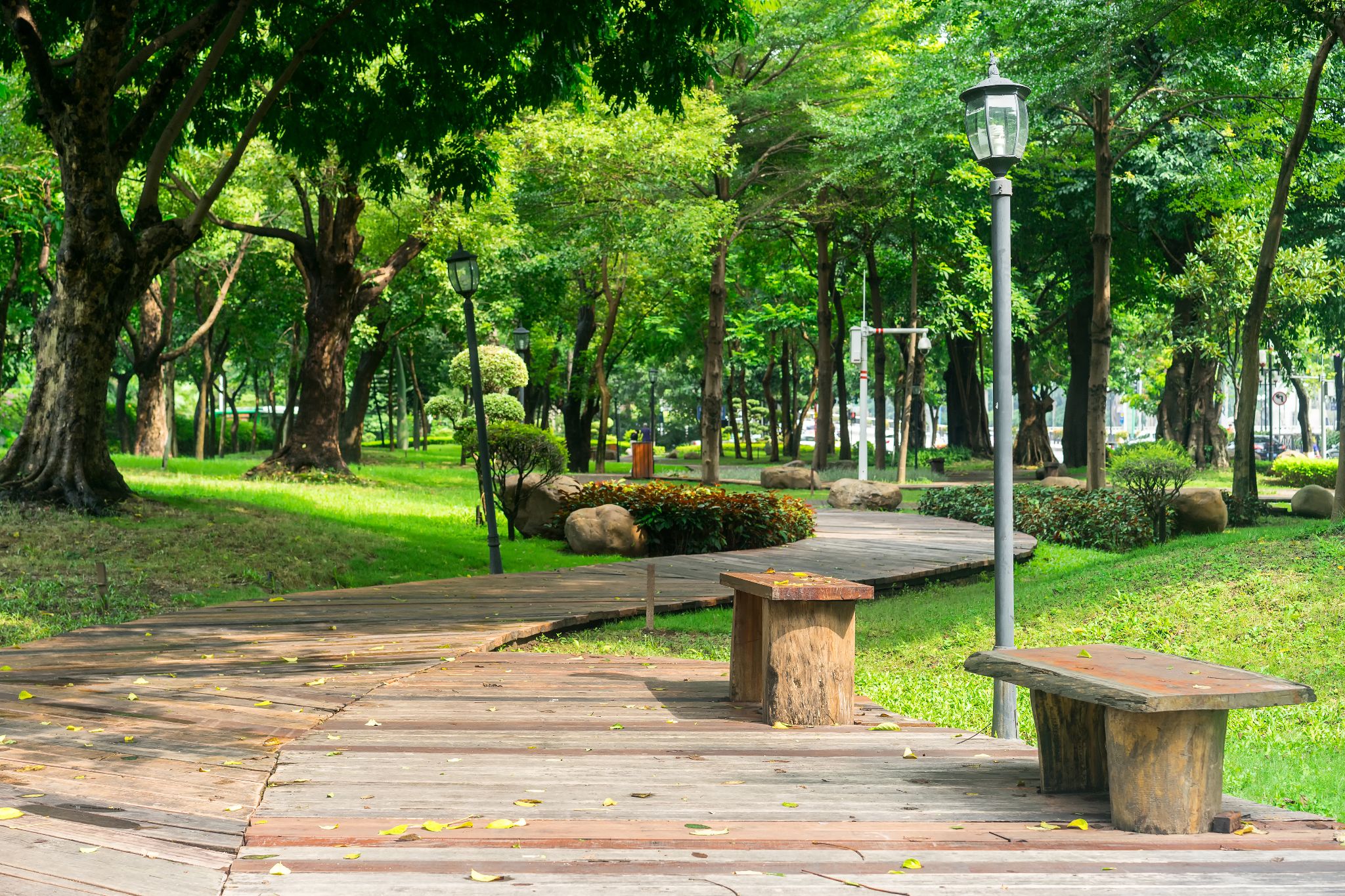


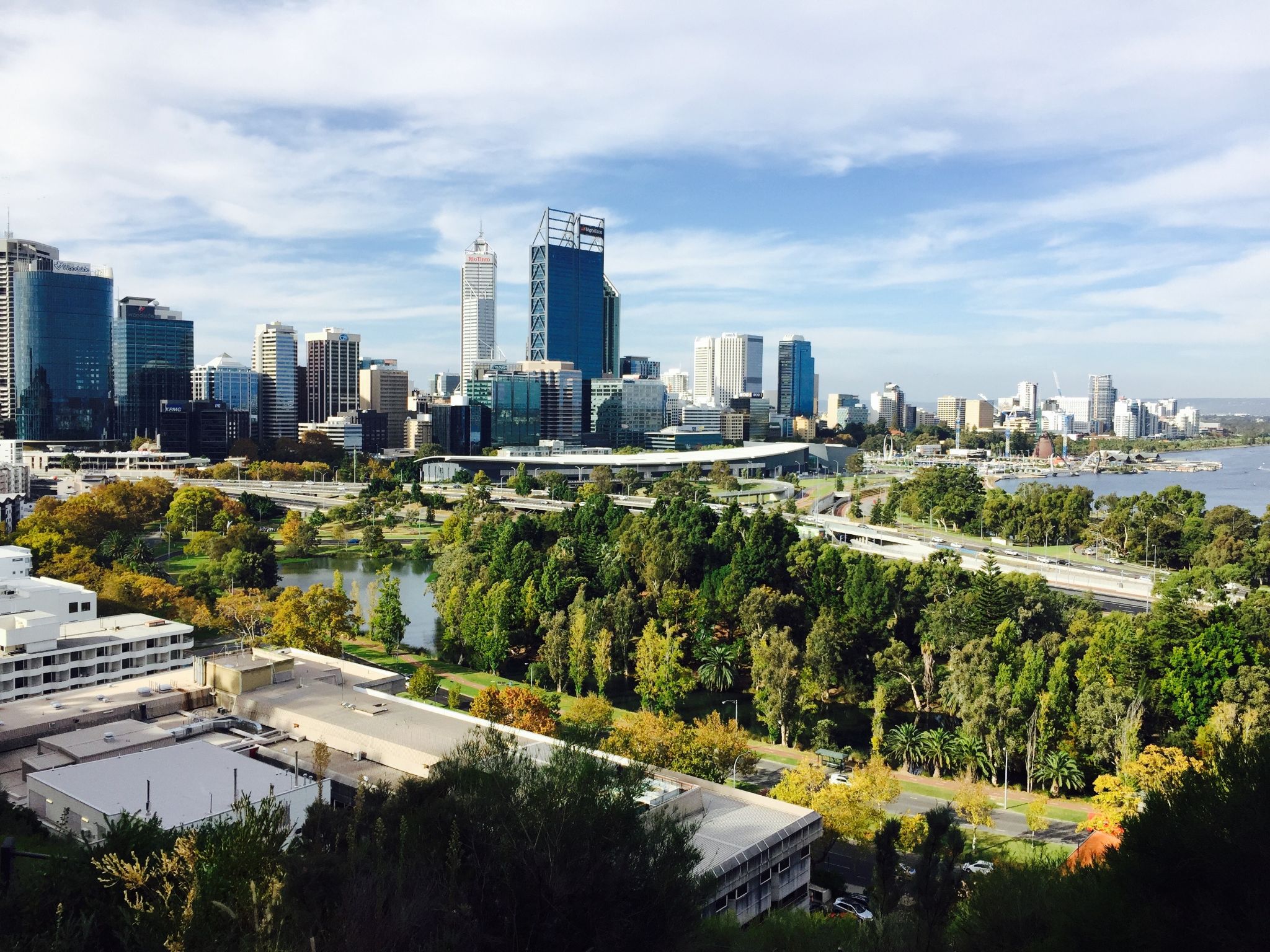


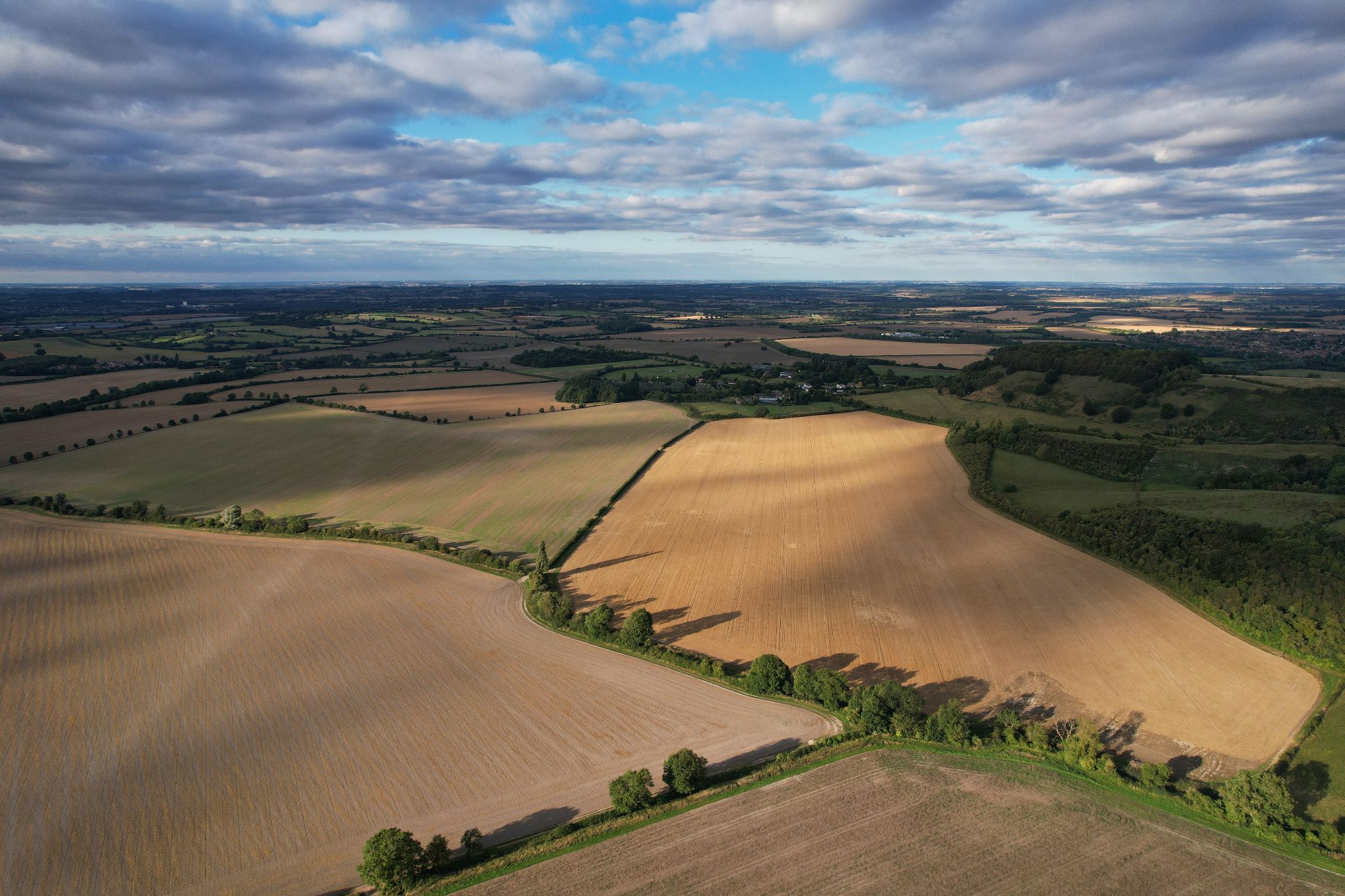


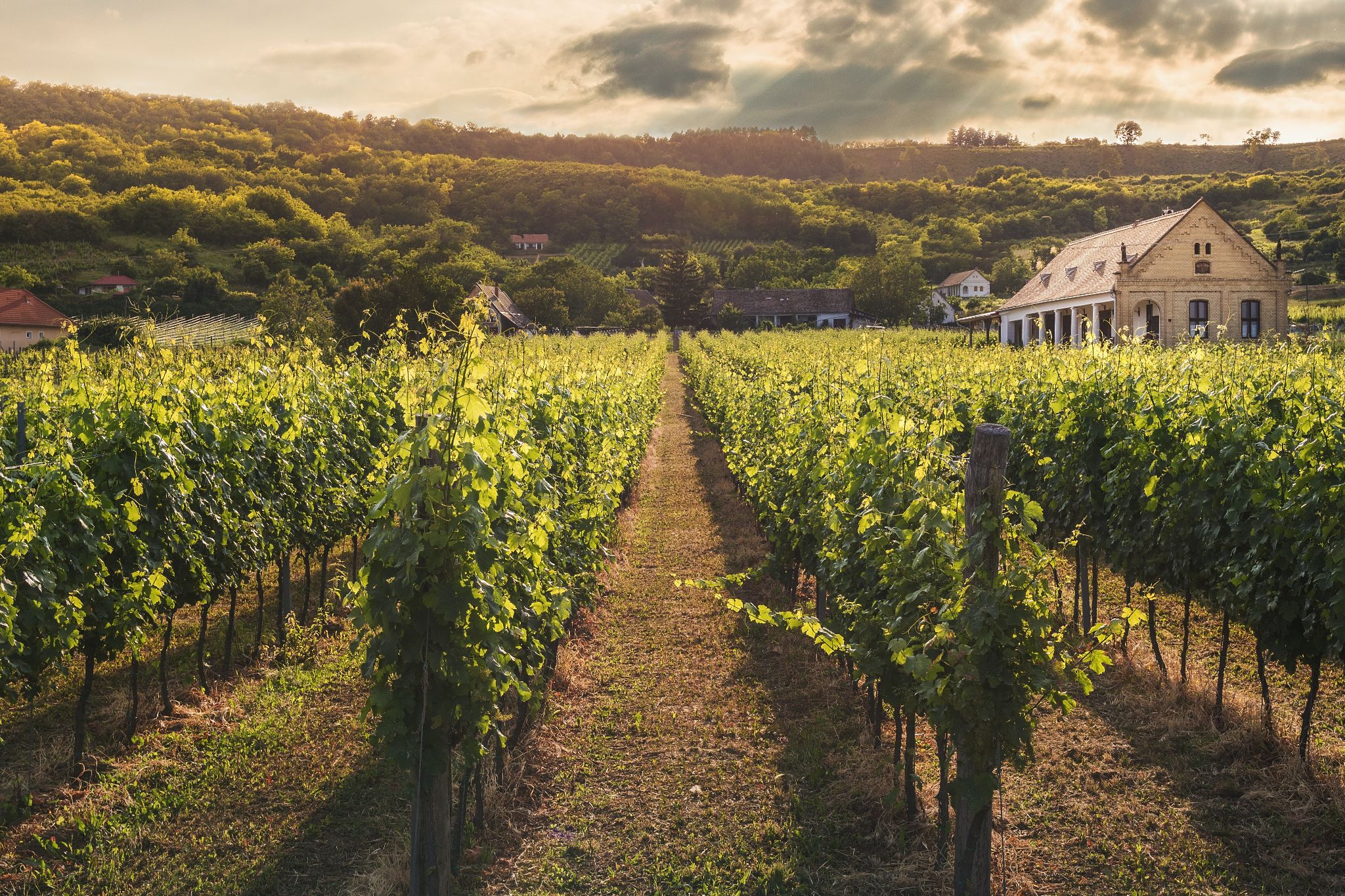

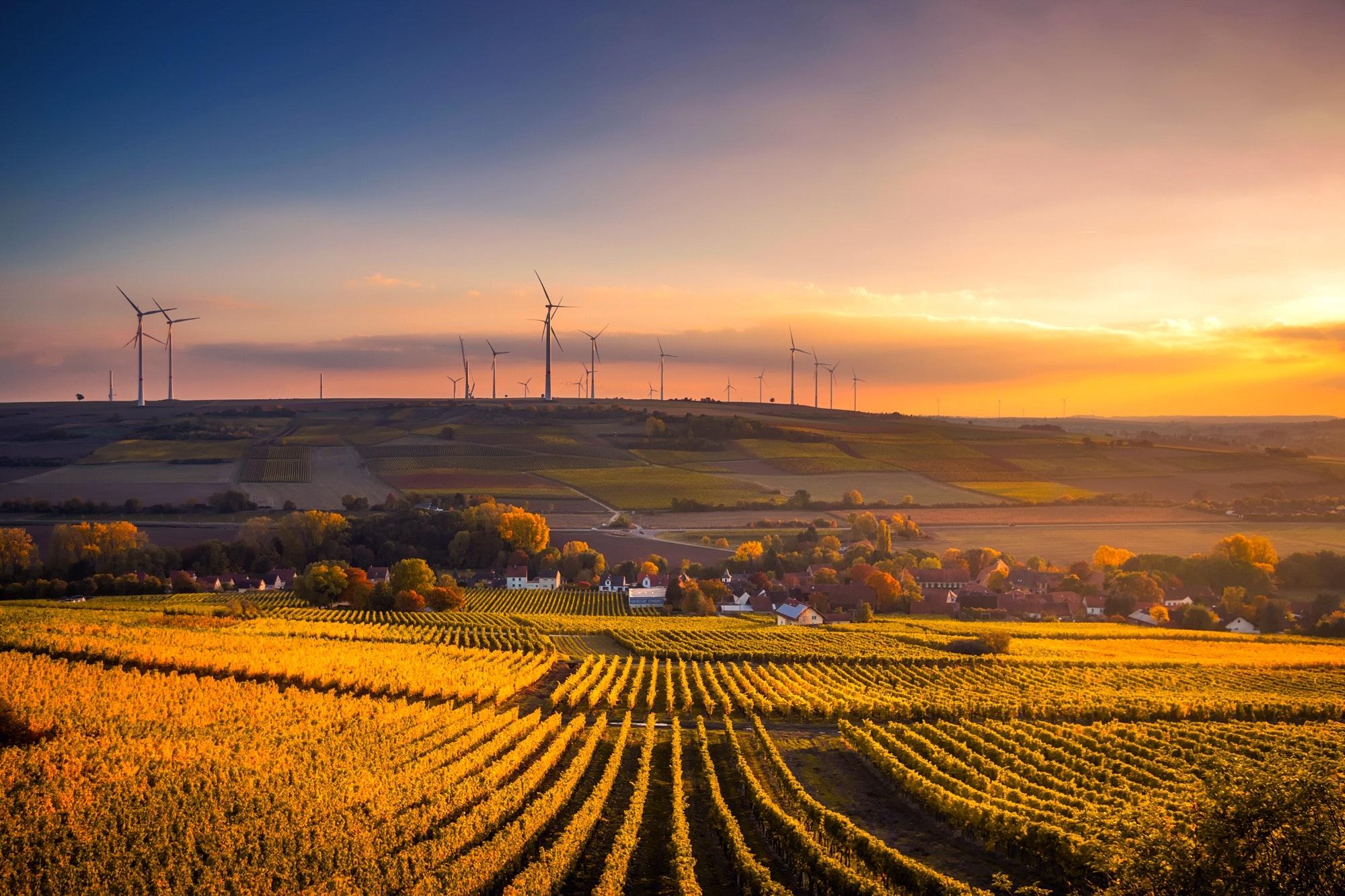


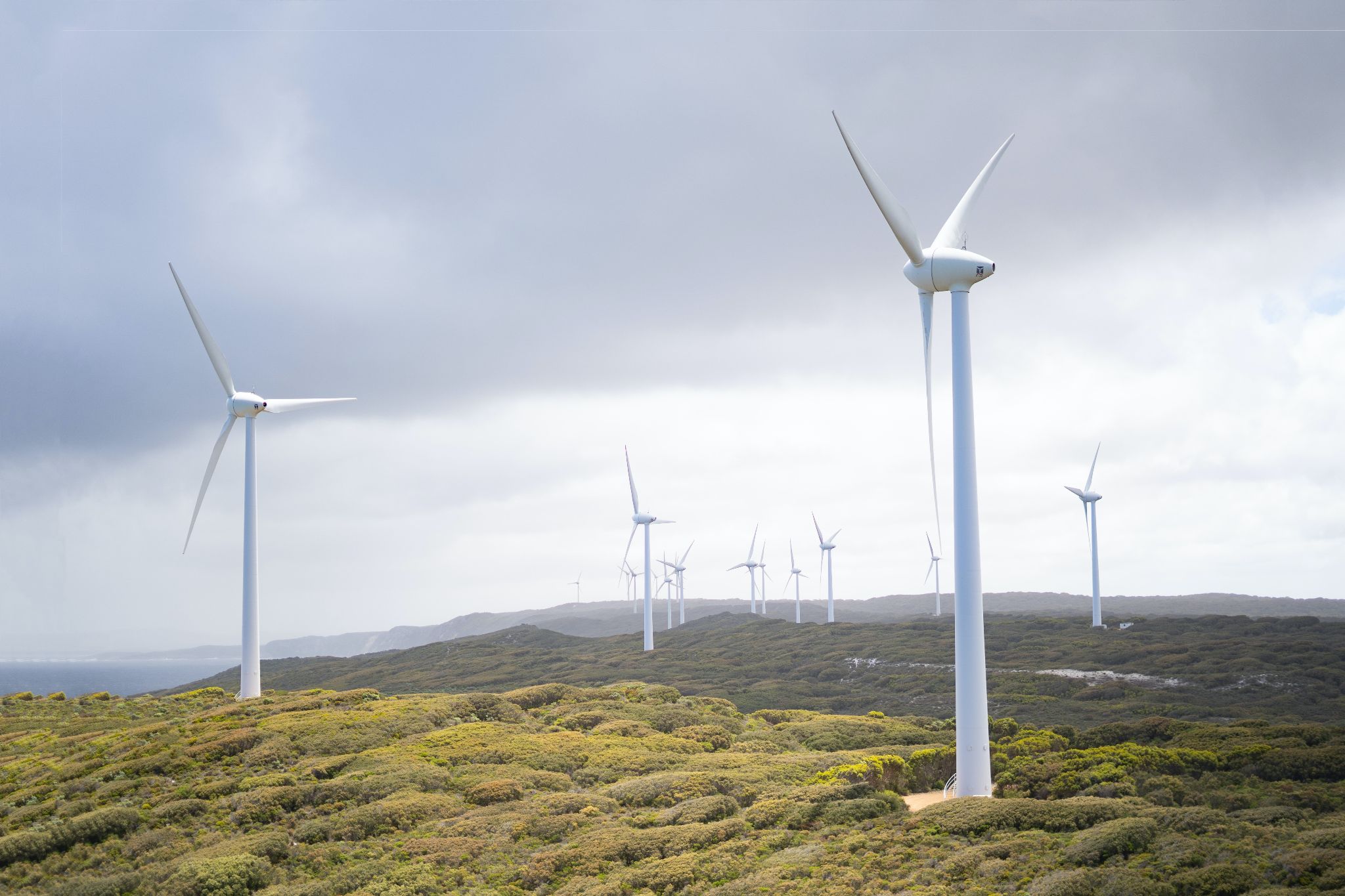


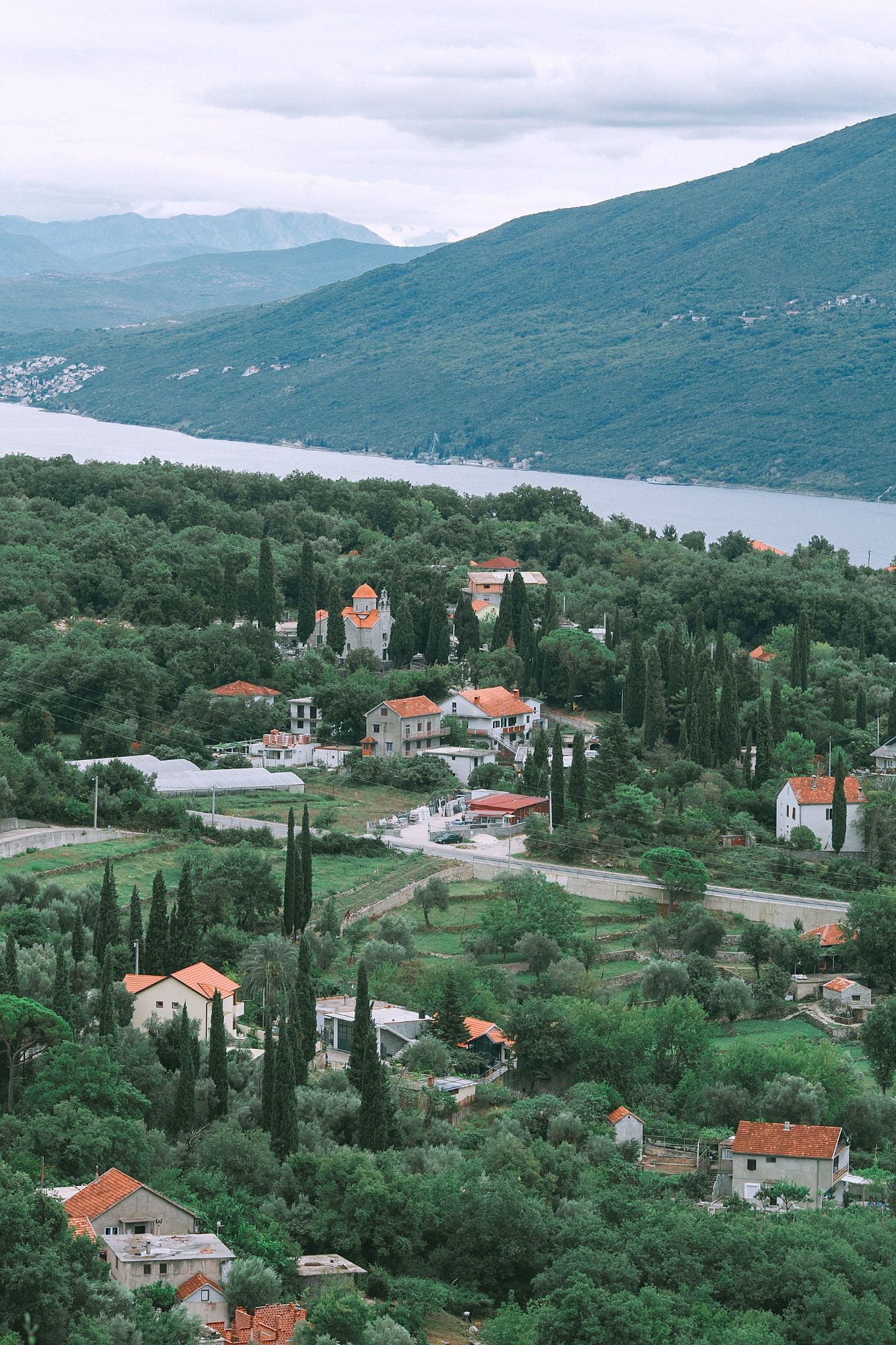


**Figure S2**: Pictures of different European landscapes shown on the first day of the workshop during the first round of the World Café Method. © Pexels

We specifically adopted the World Café engagement method, a structured technique to foster open dialogue and exchange of various ideas, allowing people to move among tables of conversation (Brown, 2010). It generally starts creating a welcoming and relaxed atmosphere, resembling a café, with small tables. Participants gather at tables to discuss a specific question or topic for about 20-30 minutes per round. After each round, they move to different tables, with one "table host" staying behind to summarise the previous conversation for the new group (Brown, 2010). In this case, we organised people into three groups, each corresponding to one of the NF corners and arranged multiple rounds of 30-minute conversation. Afterwards, each participant moved among tables, contributing to different discussions covering all aspects of the three corners of the NFF triangle. Since the NFF and related discussion may be something difficult to understand for people not familiar with it, we decided to keep an internal expert as a moderator at each table to facilitate the conversation and guide the dialogues. The moderators also prompted the discussion with the questions and took notes of the answers. In addition, to make each person actively involved in the discussion we provided boards, post-it notes and visual items. In this way, even if one person did not take the floor during the discussion, we ensured that written ideas and reflection were collected and incorporated in the outcomes.

During the afternoon session on Day 1, attendees shared their perspectives on the themes previously identified (Appendix 2.1). Employing the World Café method again, the session facilitated multiple rounds of conversation, enabling participants to self-organise and engage in discussions aligned with their interests around five tables, one per each topic.

On Day 2, the focus was on connectivity. The initial discussion utilised the World Café format with six tables, featuring two tables per NF corner, and it was facilitated by moderators who took notes on participants’ answers to specific questions. Each table addressed three primary questions: 'Why is connectivity important in this NF?', 'What are the main threats to preserving and enhancing connectivity?', and 'What species, ecological processes, and/or ecosystem services should be prioritised?' Participants rotated between tables every 20 minutes, engaging in diverse conversations across different corners. A subsequent set of questions included: 'What type of areas do we need to connect in this NF?', 'Where should we allocate connectivity?', and 'What will be the impact of developing other infrastructures?'.
 Following this step, a collective brainstorming session was conducted to emphasise priorities, enablers, and obstacles in implementing connectivity. Participants documented their ideas on five distinct boards, each associated with one policy framework or activity sector: Green and Blue Infrastructure, habitat conservation and ecosystem restoration, agroecological policies, infrastructure development and renewable energies, and species conservation. Participants were given the autonomy to choose their own topic and time for discussion.
 Day 3 delved into aspects of protected areas planning through two rounds of conversation. Participants discussed the same set of questions in five tables together with moderators, aiming to highlight differences among the three value perspectives. The four main questions included: 'Where should strict protection take place and why, in each NF?', 'What should take priority in each NF in terms of identifying and managing the rest of the Protected Area (PA) network?', 'Where and why would you allow some human activities inside PAs?', and 'Where and why would you have larger or smaller PAs in each NF corner?)’. During the afternoon of Day 2 and Day 3, discussions were not addressed towards the three NFF perspectives.

Immediately after the workshop, the notes taken by each moderator were collected, cleaned up and organised in a shared document, based on the days of the workshop and the topic they referred to. Thus, the folder with all the notes was shared to the participants, who were asked to add further input if considered worthwhile.

#### Appendix 2.3 Elaboration of draft narratives based on the workshop outcomes and formulation of further specific questions

Shortly after the workshop, a series of meetings and interviews were conducted with all moderators to ensure a comprehensive understanding of the discussions at each table and develop the first set of the narratives (**step 4**) . The goal was to further refine the notes gathered during the workshop. Subsequently, all the inputs provided by participants were thoroughly checked and organised according to the NFF corners. In addition, we streamlined the narratives, narrowing down the focus to a concise selection of recurring topics, including Urban systems, Forest Ecosystems, Freshwater Ecosystems, and Energy. We also synthesised agro-ecological policies under the main topic of Agriculture, and grouped the other themes and the outcomes of Day 2 and Day 3 under Nature Protection and Restoration. Thus, we focused the narratives on this smaller set of six topics. By doing the analysis, gaps and inconsistencies among the narratives emerged, particularly on the preferences related to Nature and Restoration, and Agriculture. To gather feedback and obtain additional insights on the current description of these narratives, a review process was initiated, together with the NaturaConnect consortium. We formulated further specific questions to be asked during a second consultation event (**step 5**):

1. In a Nature for Nature scenario, what activities would you restrict in strictly protected areas?
2. In a Nature for Society scenario, what activities would you restrict in strictly protected areas?
3. In a Nature as Culture scenario, what activities would you restrict in strictly protected areas?
4. In a Nature for Nature scenario, what kinds of forestry activities would be allowed?
5. In a Nature for Society scenario, what kinds of forestry activities would be allowed?
6. In a Nature as Culture scenario, what kinds of forestry activities would be allowed?
7. In a Nature for Nature scenario, what types of agricultural land uses should be promoted?
8. In a Nature for Society scenario, what types of ecosystem services can be reinforced in agricultural landscapes?
9. In a Nature as Culture scenario, what cultural landscapes are important for nature conservation?
10. How important is the reduction of agricultural land in each scenario?
11. In which scenario(s) do you think high-density urban areas should be emphasised?
12. Which green elements should be integrated into which scenarios?
13. In a Nature for Nature scenario, in which areas and ecosystems should we implement large scale rewilding?
14. In a Nature for Society scenario, where could ecological corridors be prioritised?
15. In a Nature as Culture scenario, what measures can contribute to improve Green Infrastructure?

#### Appendix 2.4 Refinement of the final narratives version: second elicitation stage

A 2-hour online public webinar titled 'Nature Future Scenarios for a Resilient Trans-European Nature Network (TEN-N)' was held on July 4, 2023 (**step 6**). The event was mainly advertised online by the NaturaConnect consortium, via websites and social media. 115 people joined the webinar, however it was not conclusively possible to determine their field of expertise, since only 60% participants answered the questions about the sector they belong to. The draft narratives for each topic were introduced by scientists from the NaturaConnect consortium, highlighting the contrasting perspectives within the three narratives. Following each presentation, participants were engaged in Q&A sessions, responding via Mentimeter (<https://www.mentimeter.com/>). The Mentimeter questions, designed to gather additional input and address gaps that were identified in the in-person workshop, aimed to enhance the narratives. Post-webinar, the collected responses were analysed, identifying recurring statements and integrating participant feedback into the narratives (**step 8**). The entire webinar, including discussions and presentations, was recorded and made publicly accessible as an online resource.

The second version of the narratives were reviewed by the NaturaConnect consortium member and moderators of the elicitation events (**step 8**). This step ensured that the statements in the revisited narratives relied on the initial inputs and were realistic within the European context.

Finally, we integrated the reviews and produced the final version of the narratives (**step 9**).

## References

Brown, J. (2010). The World Café: Shaping our future through conversations that matter. [www.ReadHowYouWant.com](http://www.readhowyouwant.com) accessed on 27th October 2023.

EC (2016) No net land take by 2050? European Commission, Directorate-General for Environment, Publications Office of the European Union, 2016. <https://data.europa.eu/doi/10.2779/537195>.

EC (2020a). EU biodiversity strategy for 2030: bringing nature back into our lives. Communication from the commission to the european parliament, the council, the european economic and social committee and the committee of the regions. European Commission. COM 380. Brussels. <https://data.europa.eu/doi/10.2779/048>

EC (2022a). Proposal for a Regulation of the European Parliament and of the Council on Nature Restoration. Brussels, 22.6. 2022, COM 304 Final 2022/0195 (COD).

EC (2023b). European Climate Law. European Commission. <https://climate.ec.europa.eu/eu-action/europeangreen-deal/european-climate-law_en> accessed on 10th November 2023.

EEA (2020). Building a coherent Trans-European Nature Network, 05/2020, European Environment Agency.
